# whoishRisk – an R package to calculate WHO/ISH cardiovascular risk scores for all epidemiological subregions of the world

**DOI:** 10.12688/f1000research.9742.2

**Published:** 2017-03-08

**Authors:** Dylan Collins, Joseph Lee, Niklas Bobrovitz, Constantinos Koshiaris, Alison Ward, Carl Heneghan

**Affiliations:** 1 Nuffield Department of Primary Care Health Sciences, University of Oxford, Oxford, UK

**Keywords:** WHO/ISH, Cardiovascular Risk Charts, Risk Score, R

## Abstract

The World Health Organisation and International Society of Hypertension (WHO/ISH) cardiovascular disease (CVD) risk assessment charts have been implemented in many low- and middle-income countries as part of the WHO Package of Essential Non-Communicable Disease (PEN) Interventions for Primary Health Care in Low-Resource settings. Evaluation of the WHO/ISH cardiovascular risk charts and their use is a key priority and since they only existed in paper or PDF formats, we developed an R implementation of the charts for all epidemiological subregions of the world. The main strengths of this implementation are that it is built in a free, open-source, coding language with simple syntax, can be downloaded from github as a package (“whoishRisk”), and can be used with a standard computer.

## Introduction

Cardiovascular disease (CVD) is the leading cause of death worldwide, including in many low-and-middle income countries (LMIC) ^1, 2^. Preventing CVD is therefore a worldwide priority and the World Health Organisation (WHO) is coordinating a global strategy for LMIC to systematically prevent CVD in primary care ^3^.

In 2007 the WHO and the International Society of Hypertension (ISH) published the WHO/ISH CVD risk charts for all WHO epidemiological subregions of the world ^4^. These charts are to be used as part of the WHO’s Package of Essential NCD (PEN) Interventions for Primary Health Care in Low-Resource Settings in jurisdictions that do not have their own population-derived risk assessment algorithms. While these charts are a good resource for many health systems, little is known about their validity ^5^. Therefore, it is important that jurisdictions that implement these charts conduct operational research and attempt to validate and optimise them for their setting.

Two paper-based versions of WHO/ISH charts are available for each subregion: one that requires measured total cholesterol and one that does not. The latter was made available for use in settings with limited access to laboratory testing or where the cost of cholesterol testing is prohibitive. Both charts require information on age, gender, diabetes status, smoking status, and systolic blood pressure to stratify people into one of five risk categories of 10-year risk of a fatal or non-fatal CVD event. Further instructions for their use have been published, including the definition and classification of the fourteen epidemiological sub-regions ^3^.

Through our experience collaborating with LMIC with the implementation of WHO PEN, we identified a common need for an open-source tool to facilitate the implementation of WHO/ISH risk charts and operational research of WHO PEN at a population level. We therefore developed an R package called whoishRisk, which we describe here and make available to researchers in LMIC. R is a statistical computing language and environment which is open source and freely available to anyone ^6^.

## Methods

### Extraction of WHO/ISH cardiovascular risk charts

We extracted all versions of the paper-based WHO/ISH CVD risk charts by hand into a standardized Microsoft Excel template, independently and in duplicate. We compared the duplicate extractions and calculated Cohen’s kappa coefficient for inter-rater reliability, using the irr package in R ^7^. Discrepancies were reviewed by the same two extractors and resolved by referring to the original paper chart.

### Development of the WHO/ISH risk function

One author wrote the initial code for the WHO/ISH risk function in R and created the whoishRisk package (DC). This was reviewed and adapted by a second author experienced in the R language (CK). Two additional authors (JL, NB), new to the R language, reviewed the code to ensure the syntax was comprehensible.

### Validation

A MatLab implementation of WHO/ISH risk charts for epidemiological subregion SEAR D had been previously reported ^8^. We used Octave ( www.gnu.org/software/octave/) version 8.3.2 to calculate the SEAR D WHO/ISH risk score for every possible combination of risk factors using the previously reported MatLab implementation, and compared the percent agreement to the risk scores generated by our R package, whoishRisk.

## Results

### whoishRisk Package

The whoishRisk package can be downloaded and installed directly from github using the install_github() command in the devtools package, with the argument “DylanRJCollins/whoishRisk” ^9^. The package contains a single function, WHO_ISH_Risk(), which calculates the WHO/ISH CVD risk score for any epidemiological subregion of the world based on the parameter values passed to it.

### Extraction of WHO/ISH cardiovascular risk charts

All WHO/ISH risk charts were extracted by hand into a single comma delimited file ( Dataset 1). The first six columns specify the risk factor values, and the last 14 columns specify the corresponding risk category for a given subregion. whoishRisk uses these data internally to calculate the WHO/ISH risk score. Cohen’s kappa for initial agreement between the independent extractors was 0.97, indicating excellent agreement. All remaining discrepancies were resolved by consensus.

WHO_ISH_Scores.csv CSV file of all parameter combinations and corresponding WHO/ISH risk scores used internally by WHO_ISH_Risk() to match parameter values passed to it to their respective risk score. Click here for additional data file. Copyright: © 2017 Collins D et al. 2017 Data associated with the article are available under the terms of the Creative Commons Zero "No rights reserved" data waiver (CC0 1.0 Public domain dedication).

### Development of the WHO_ISH_Risk() Function

whoishRisk contains a single function, called WHO_ISH_Risk(), which calculates the WHO/ISH risk score for any epidemiological subregion. The function code is reported herein ( Dataset 2).

R code for the WHO_ISH_Risk() function of the whoishRisk package Click here for additional data file. Copyright: © 2017 Collins D et al. 2017 Data associated with the article are available under the terms of the Creative Commons Zero "No rights reserved" data waiver (CC0 1.0 Public domain dedication).

WHO_ISH_Risk() requires seven parameters: age, gender, smoking status, diabetes status, systolic blood pressure, total cholesterol, and the appropriate WHO epidemiological subregion. The function format in the workspace is: WHO_ISH_Risk(age, gdr, smk, sbp, dm, chl, subregion). These parameters and their abbreviations are summarised in Table 1. No default values are specified for any parameter.

**Table 1.** Description of the WHO_ISH_Risk() function parameters.

Function parameter	Full parameter name	Parameter class	Parameter values
age	Age	numeric	Continuous (years)
gdr	Gender	numeric	Dichotomous (0=female; 1=male)
smk	Smoking	numeric	Dichotomous (0=non-smoker; 1=smoker)
sbp	Systolic blood pressure	numeric	Continuous (mmHg)
dm	Diabetes mellitus status	numeric	Dichotomous (0=not diabetic; 1=diabetic)
chl	Total cholesterol	numeric	Continuous (mmol/ L); 0=unknown cholesterol
subregion	WHO epidemiological subregion	character	“AFR_D”, “AFR_E”, “AMR_A”, “AMR_B”, “AMR_D”,“EMR_B”, “EMR_D”, “EUR_A”, “EUR_B”, EUR_C”, “SEAR_B”, “SEAR_ D”, “WPR_A”, “WPR_B”

Internally, WHO_ISH_Risk() requires access to the comma delimited file named “WHO_ISH_Scores.csv”, which it calls automatically from within the package but is also included herein ( Dataset 1).

The WHO_ISH_Risk() function creates an internal data frame of the risk factor values passed to it. Parameters can be single values or vectors of equal length. It then categorises the continuous parameters age, systolic blood pressure, and total cholesterol. Age and systolic blood pressure were categorised according to WHO guidance ^10^. Total cholesterol was categorised into one of the five possible categories (<=4,5,6,7 and >=8 mmol/L) according to common clinical practice, rounding up from 0.5 to the nearest integer.

Internally, a unique identification code is generated corresponding to the combinations of risk factors for each individual. This code is matched to a reference code from the “WHO_ISH_Scores.csv” file. The function stores the risk scores in a data frame that includes the risk factors, and ultimately returns a vector containing the risk scores. The output of the function is one of five different character strings, corresponding to the five different WHO/ISH risk categories: “<10%”, “10 to <20%”, “20 to <30%”, “30 to <40%”, >=40%”.

Warning messages are included when parameters appear out of range. These messages, their conditions, and their intended interpretation are described in Table 2. Out of range continuous parameters (age, systolic blood pressure, total cholesterol) are non-fatal and a risk score will be generated with a warning message. Out of range dichotomous variables (gender, smoking status, diabetes status) are fatal errors and the output (NA) will be generated with a warning.

**Table 2.** WHO_ISH_Risk() warning messages, their conditions, and intended interpretation.

Condition of Warning	Warning Message	Interpretation of Warning
age < 19	"At least one age is 18 or younger"	WHO/ISH risk scores are meant for use in adults. At least one age value is equal to 18 or younger. Check for errors in age values.
age >100	"At least one age is greater than 100"	Age values greater than 100 may indicate a data error. Check to ensure age values over 100 are correct.
gdr > 1	"Gender must be equal to 0 or 1"	A value for gender other than 0 or 1 is included. Check gender values.
smk > 1	"Smoking must be equal to 0 or 1"	A value for smoking other than 0 or 1 is included. Check smoking values.
sbp < 90	"At least one systolic blood pressure is below 90 mmHg"	SBP values below 90 mmHg may indicate a data error. Check to ensure values under 90 mmHg are correct.
sbp > 250	"At least one systolic blood pressure is over 250 mmHg"	SBP values over 250 mmHg may indicate a data error. Check to ensure values over 250 are correct.
dm > 1	"Diabetes status must be equal to 0 or 1"	A value for diabetes status other than 0 or 1 is included. Check diabetes status values.
tc > 10	"At least one total cholesterol is greater than 10 mmol/L. Ensure all values are in units of mmol/L"	Total cholesterol values over 10 mmol/L may indicate a data error. Check to ensure values over 10 mmol/L are correct and in units of mmol/L.

### Worked example

whoishRisk can be installed in one step using install_github() from the devtools package ^9^. We have included a worked example of how to install whoishRisk and use the WHO_ISH_Risk() function to calculate the risk score for five individuals.

#Step 1: Install whoishRisk package
> library(devtools)
> install_github("DylanRJCollins/whoishRisk")
#Step 2: Load whoishRisk package into workspace
> library(whoishRisk)
#Step 3: Load risk factor data
> Age <- c(40, 87, 65, 53, 71) #Age in years
> Gender <- c(0,0,0,1,1) #0=female, 1=male
> Smoking <- c(1,1,0,1,0) #0=non-smoker 1=smoker
> Systolic_Blood_Pressure <- c(129, 157, 134, 189, 141) #SBP in mmHg
> Diabetes <- c(1,1,1,0,1) #0=not diabetic 1=diabetic
> Total_Cholesterol <- c(0, 5.1, 4.5, 0, 8.3) #Total cholesterol (mmol/L, 0=unknown cholesterol)
#Step 4: Pass the risk factor vectors to the WHO_ISH_Risk() function, and set subregion equal to the name of the appropriate epidemiological subregion (e.g. “EMR_B”). This will return a vector of WHO/ISH risk scores.
> WHO_ISH_Risk(Age, Gender, Smoking, Systolic_Blood_Pressure, Diabetes, Total_Cholesterol,"EMR_B")
[1] "<10%" ">=40%" "<10%" ">=40%" ">=40%"

## Validation

Comparison with the published MatLab implementation of the SEAR D risk charts ^8^ showed 100% agreement with our R implementation, for all possible combinations of risk factors.

## Discussion

To our knowledge, this is the first publically available R implementation of WHO/ISH CVD risk charts for all WHO epidemiological subregions of the world. Our package, whoishRisk, may be used for analysis of cardiovascular risk when electronic patient data is available. The code will automatically apply WHO/ISH risk scores to patients based on age, gender, systolic blood pressure, smoking status, diabetes status, total cholesterol, and epidemiological subregion. This code could be used, for example, during a pilot implementation of WHO PEN to audit the accuracy of risk assessment by comparing documented risk scores to actual risk scores calculated using this tool. We have provided a complete worked example.

While WHO PEN guidance specifies the range of systolic blood pressure values for each systolic blood pressure category, it provides no such guidance for categorising total cholesterol. Based on our opinion and clinical experience, and on a previously published implementation in MatLab ^8^, we chose to categorise total cholesterol by rounding up at 0.5 to the next integer.

The “WHO_ISH_Scores.csv” file is provided herein for transparency and to promote collaboration and cross validation. While the risk score values it stores are returned to the workspace as characters (e.g. “10 to <20%”), a user could simply convert these to class numeric or factor. We chose to return them as character strings that are identical to the patient charts in order to produce a literal implementation of the risk charts.

## Conclusion

We created an R package called whoishRisk to be used for the calculation of WHO/ISH CVD risk charts for all WHO epidemiological subregions of the world. It contains a single function, WHO_ISH_Risk(), that requires seven parameters: age, gender, systolic blood pressure, smoking status, diabetes status, total cholesterol, and epidemiological subregion. whoishRisk can be used to quickly calculate WHO/ISH risk scores from routinely collected electronic patient data and therefore aid in the implementation and evaluation of these risk charts in low-resource settings.

## Data and software availability

*F1000Research*: Dataset 1. CSV file of all parameter combinations and corresponding WHO/ISH risk scores used internally by WHO_ISH_Risk() to match parameter values passed to it to their respective risk score 10.5256/f1000research.9742.d153375 ^11^

*F1000Research*: Dataset 2. R code for the WHO_ISH_Risk() function of the whoishRisk package, 10.5256/f1000research.9742.d153376 ^12^
