# Supplementary material for: whoishRisk – an R package to calculate WHO/ISH cardiovascular risk scores for all epidemiological subregions of the world
Source: F1000Res. 2017 Mar 8;5:2522. Originally published 2016 Oct 14. [Version 2] doi: 10.12688/f1000research.9742.2 (PMC5345772; doi:10.12688/f1000research.9742.2)
Supplement: Peer review history

# Peer review: Referee response for version 2

**DOI:** 10.5256/f1000research.11940.r20822

**Published:** 2017-03-14

**Authors:** Scott A. Chamberlain

**Affiliations:** 1 rOpenSci project, University of California, Berkeley, Berkeley, CA, USA

I'm mostly satisfied with the author's changes, with a few minor changes. 

 Thank you for making an R package. I downloaded, and checked the package, with a few observations:

- In the package use utils::read.csv instead of just read.csv - you probably saw the warning if you've run R CMD CHECK or devtools::check() 
- The package has no examples. I'd strongly suggest including examples.
- The package has no tests. I'd strongly suggest including tests (though if time is limited, at least include examples).
- The authors note that the package now gives warnings when input values are out of range - I'd strongly suggest to document what the correct ranges are for each parameter so users don't have to guess
- In addition, if there are sensible default values I'd use those - if applicable - may not be applicable. 
- Urge authors to document what type of value is expected for each parameter, e.g., age (numeric), subregion (character)

Sincerely,

 Scott Chamberlain

I have read this submission. I believe that I have an appropriate level of expertise to confirm that it is of an acceptable scientific standard.

# Peer review: Referee response for version 2

**DOI:** 10.5256/f1000research.11940.r20821

**Published:** 2017-03-10

**Authors:** Maria Suarez-Diez

**Affiliations:** 1 Laboratory of Systems and Synthetic Biology, Wageningen University and Research (WUR) , Wageningen, Netherlands

My main concerns have been addressed in this new version of the manuscript.

 I think the developed package represents a useful addition to the field. I was able to install and run the package and indeed the package reports the absence of data points (NA).

 Only a minor concern: within R, getting the citation 'citation("whoishRisk")' returns the names of the name of the package, the names of the authors and the R version. I recommend they include a reference to this work.

I have read this submission. I believe that I have an appropriate level of expertise to confirm that it is of an acceptable scientific standard.

# Peer review: Referee response for version 2

**DOI:** 10.5256/f1000research.11940.r20823

**Published:** 2017-03-09

**Authors:** Raivo Kolde

**Affiliations:** 1 Philips Research North America, Cambridge, MA, USA

All my main concerns were satisfied with the update. I was able to install and run the package. I guess from visibility standpoint it would be nice to include it to the CRAN database, but otherwise I approve. 

I have read this submission. I believe that I have an appropriate level of expertise to confirm that it is of an acceptable scientific standard.

# Peer review: Referee response for version 1

**DOI:** 10.5256/f1000research.10503.r17004

**Published:** 2016-10-28

**Authors:** Maria Suarez-Diez

**Affiliations:** 1 Laboratory of Systems and Synthetic Biology, Wageningen University and Research (WUR) , Wageningen, Netherlands

The authors present an R script and an additional datafile to calculate cardiovascular risk using the Who/ISH risk assessment charts. Previous implementations required the use of MatLab and I believe an R implementation can be an useful addition to the field.

 The authors intended to design a code requiring little R expertise. I think the authors have only partly succeeded in accomplishing this goal and the code still needs to be improved.

 As mentioned by the other reviewers, RTF is far from an optimal format to distribute the code. The authors should present their code as a package hosted in some repository. Currently the RTF file contains the function definition, data loading and working example in a single view. When creating the package the authors should clearly separate the code from the documentation and provide a working example. If the authors intent this tool to be used and possibly modified by users with little R expertise then a separate file with detailed instruction should be provided.

 The authors state that users can modify the WHO_ISH_Scores.csv file to represent other risk categories. That would require explanation of the content of the file. Although the used abbreviations gdr for gender might seem obvious, they should be explained. Also when downloading the file from F1000, the file name is changed and appears as “5ae9107XXX..._WHO_ISH_Scores.csv” so that the reading fails. This could be fixed by including the file as a dataset in the corresponding package.

 When data from multiple patients are provided (as in the example) the output is a factor. I think it would help the users match input and output values is some tabular format or some patient identifier is provided in the output.

 If any of the input parameters are missing (NA values) the code outputs “NA” but no information regarding the missing values is provided. It might be helpful if the code were to report which value was actually missing.

 Fig. 1 shows the code and contains mainly information on how some continuous variables (age, cholesterol and systolic blood pressure are discretized). I think a more efficient way of conveying this information is by including it in Table 1.

I have read this submission. I believe that I have an appropriate level of expertise to confirm that it is of an acceptable scientific standard, however I have significant reservations, as outlined above.

# Response

**Published:** 2017-03-06

**Authors:** Dylan Collins

**Affiliations:** University of Oxford, UK

Dear Maria Suarez-Diez,

 Thank you for your valuable comments. In response we have made the following changes:

- We created an R package including all documentation files which can be downloaded from github
- We no longer suggest users to change the “WHO_ISH_Scores.csv” files and we have updated the file name hosted by F1000 to be “WHO_ISH_Scores.csv”. This file, as suggested, is internal to the package and while we report it here is not necessary to download.
- We have added a series of warning messages to help explain outputs of “NA” and to help catch errors in the input values (e.g. out of range parameters)
- We have described how continuous variables are discretized in the main text

Every Best, 

 Dylan Collins

# Peer review: Referee response for version 1

**DOI:** 10.5256/f1000research.10503.r17006

**Published:** 2016-10-25

**Authors:** Scott A. Chamberlain

**Affiliations:** 1 rOpenSci project, University of California, Berkeley, Berkeley, CA, USA

The authors describe some R code for helping to calculate cardiovascular risk scores. I think the code needs significant work.  

- The code is in an .rtf file. This is very bad software practice. The authors should at the very very least put the code in a file with .R extension.  
- Ideally the code should be put in an R package. It is relatively straight-forward to make an R package these days. See http://r-pkgs.had.co.nz/ for help. This makes it easy to add documentation (of which there is currently essentially none for the current function included in the manuscript), tests, etc. Looks like the authors have a Github repository (https://github.com/DylanRJCollins/WHO_ISH_R_Implementation) - this could be made into an R package. 
- Another benefit of making a package, is that R packages have a way of including datasets in them. The csv file is small enough that you can include it in the package if that's appropriate for the use case here. If the `WHO_ISH_Scores.csv` dataset is not likely to change, or not likely to change very often, they can include it in the package.
- The dataset `WHO_ISH_Scores.csv` has two columns that are probably row names that should be removed. If they aren't data, remove them. 
- The dataset `WHO_ISH_Scores.csv`: the data is not in a tidy format that is easy to work with. There shouldn't be numeric data combined with symbols (e.g. >=40%). If possible, I urge authors to find a way to make these into numeric columns while retaining the same information. However, it may be that it's too difficult to separate numeric values from the greater than / less than symbols etc.
- R packages should be cited by reference. e.g. "using the irr package (version 0.84)". It is good they cite the version, but put a reference in in your references. Run `citation("irr")` in an R session to get a reference for it. 
- "We used RStudio (version 0.99.489) to compare the duplicate extractions ...": Rstudio is just an IDE. Say that you used R, not RStudio. It's fine to cite RStudio, but also cite R. Run `citation()` in an R session to get the citation for R. 
- Octave is an open source language, and thus the authors should share the Octave code. 
- "[...] we intentionally sought to use simple syntax in the base package" - the authors script is in fact not a package, but if they do make a package this wording is good. 
- In the Conclusion: "We created a simple R implementation of WHO/ISH CVD risk charts for all WHO epidemiological subregions of the world, requiring only the base R package." - refer to this as "base R", not "the base R package".
- If the dataset "WHO_ISH_Scores.csv" can be modified, thus different versions of it may be used by the user, it makes more sense to pass in the dataset as a parameter, instead of hard coding reading in the data in the function. 
- I urge authors to include a license with their R package (to be created) - and to submit the package to CRAN so it's easy for all to install. 

Code comments:

- I'm not sure the authors meant to do this, but the use of single ampersand in the `ifelse` statements (e.g., `ifelse(df$age > 17 & df$age < 50, 40, df$age)`) means that they get a vector of logicals (e..g., `df$age > 17 & df$age < 50` evaluates to `TRUE FALSE FALSE FALSE FALSE`) using their example, when I think what they want is a single logical return value. If so, use double ampersand instead: `&&` instead of `&`.
- I'm pretty sure, but have not tested thoroughly, that the long series of if statements in the section "Match the look up value with the reference value" can be replaced with just this: `ref[[subregion]][match(df$luv, ref$refv)]`.

I have read this submission. I believe that I have an appropriate level of expertise to confirm that it is of an acceptable scientific standard, however I have significant reservations, as outlined above.

# Response

**Published:** 2017-03-06

**Authors:** Dylan Collins

**Affiliations:** University of Oxford, UK

Dear Scott A. Chamberlain,

 Thank you for your thoughtful and considered feedback. We have responded in the updated version as detailed below.

- We have created an R package which can be downloaded from github
- We provide the code from the R package as a .R file
- We have included the “WHO_ISH_Scores.csv” data file in the R package
- We tidied the ”WHO_ISH_Scores.csv” files but have kept the risk scores as character strings to retain a literal implementation of the risk charts, as described in the updated manuscript
- We have cited R packages and R as suggested
- The MatLab code used in Octave has previously been published as cited in the manuscript and is freely available
- We no longer suggest users to change the “WHO_ISH_Scores.csv” files
- We have included a GPL-3 license for the R Package

Every Best, 

 Dylan Collins

# Peer review: Referee response for version 1

**DOI:** 10.5256/f1000research.10503.r17007

**Published:** 2016-10-20

**Authors:** Raivo Kolde

**Affiliations:** 1 Philips Research North America, Cambridge, MA, USA

The paper describes a software tool for calculation of WHO/ISH cardiovascular risk scores for different epidemiological subregions of the world. This could be a useful piece of software for researchers working with cardiovascular epidemiological data and make the practices for calculating such scores more reproducible over multiple studies. However, in the present form I found the paper to be severely lacking in both implementation and substance.

 First, the code should not be distributed as an RTF file. R packages are well accepted standard for reproducibly distributing R code that also forces to provide some rudimentary documentation and examples. As an additional benefit, hosting the package in a repository like CRAN, Bioconductor or even Github, makes it easy to install and update the program. There is no reason to not follow this convention for this particular project, thus, the code should be converted into package format and hosted in one of the above-mentioned services to be any use to the community. 

 The argument of the code in the present form being simply adjustable is not valid. One has to spend quite a bit of time understanding what is happening in the code, especially when not an R expert. For the code to fulfill its purpose it has to be rewritten in a way that adjustable parts are presented to the user as function parameters (with reasonable defaults). This way users have to know even less R to use the code effectively. 

 Also, having a figure depicting the code is uncommon to say the least and not useful in any way. It would make much more sense to include a worked out example to the text, where you would describe a common use case of your R package.

 To enhance usefulness of the package and make it more universally applicable it would be good to see some more risk prediction models like Framingham and SCORE added to the software. This would make it easy to compare the validity of different scores over a study population.

I have read this submission. I believe that I have an appropriate level of expertise to confirm that it is of an acceptable scientific standard, however I have significant reservations, as outlined above.

# Response

**Published:** 2017-03-06

**Authors:** Dylan Collins

**Affiliations:** University of Oxford, UK

Dear Raivo Kolde,

 Thank you for your insightful review and comments. In response we have made the following changes:

- As suggested, we created an R package which can be downloaded directly from github
- We no longer advocate for the code to be adjustable and through the development of a package have made it simpler to use
- We removed the figure of the code, and have added a worked example in the text
- The main difference between scores like Framingham, SCORE, and QRISK2 is that they have underlying cox model equations which can be implemented in R, whereas WHO/ISH risk charts do not – hence the rationale for the development of this package. While the additional of further risk scores was outside the scope of this work, we welcome collaborators who might want to contribute in this respect.

Every Best, 

 Dylan Collins
